# Hereditary pseudocholinesterase deficiency in a 4-year-old girl: a case report

**DOI:** 10.1186/s13256-025-05183-5

**Published:** 2025-03-28

**Authors:** Julia Schulze-Berge, Lukas Pillong, Birgit Busse, Wolfram Henn, Nasenien Nourkami-Tutdibi, Dominik Schmitz, Tobias Hüppe

**Affiliations:** 1 Department of Anaesthesiology, Intensive Care and Pain Therapy, Saarland University Medical Centre, Homburg, Saar Germany; 2 Department of Otorhinolaryngology, Saarland University Medical Centre, Homburg, Saar Germany; 3 Medicover, MVZ Martinsried GmbH, Martinsried, Germany; 4 Department of Human Genetics, Saarland University Hospital, Homburg, Saar Germany; 5 Department of General Paediatrics and Neonatology, Saarland University Medical Centre, Homburg, Saar Germany

**Keywords:** Pseudocholinesterase deficiency, Delayed emergence, Pediatric anesthesia, Neuromuscular blockade, Case report

## Abstract

**Background:**

This report outlines a case of pseudocholinesterase deficiency in a pediatric patient, whose autosomal recessive condition is caused by two different pathologic variants of the butyrylcholinesterase gene, resulting in a rare case of functional homozygosity.

**Case presentation:**

A healthy 4-year-old girl of Northern European descent underwent general anesthesia for tonsillotomy, adenoidectomy, and bilateral tympanocentesis. Previously unknown pseudocholinesterase deficiency presented as delayed emergence with sustained apnea and paralysis following administration of mivacurium, necessitating transfer to the pediatric intensive care unit for prolonged post-operative ventilatory support and monitoring. Extubation was safely performed 8 hours later. No long-term sequelae were noted.

Genetic testing identified compound heterozygosity in the butyrylcholinesterase gene. Thus, a diagnosis of autosomal recessive hereditary pseudocholinesterase deficiency was made.

**Conclusion:**

Pseudocholinesterase deficiency will almost always present unexpectedly and must be included in the differential diagnosis of delayed emergence. Once suspected, a clinical diagnosis can be supported using a peripheral nerve stimulator, and confirmed using laboratory tests. Genetic testing can help determine the etiology of disease.

## Background

Pseudocholinesterase deficiency is an important, albeit rare, differential diagnosis of delayed emergence from general anesthesia. Insufficient enzyme activity alters the metabolism of choline esters, such as succinylcholine and mivacurium, and patients typically present with prolonged post-anesthetic apnea and paralysis [1]. Hereditary pseudocholinesterase deficiency is an autosomal recessive condition with an estimated prevalence of 1:3000 for homozygotes and 1:25 for heterozygotes [2]. It is caused by mutations in both alleles of the butyrylcholinesterase (*BChE*) gene located on chromosome 3 at 3q26.1-q26.2 [3]. Diagnostic tests for pseudocholinesterase deficiency are not part of a routine preoperative work-up, and patients are typically asymptomatic in their daily lives [4]. Consequently, a diagnosis is not usually made until an individual fails to adequately recover from neuromuscular blockade following administration of succinylcholine or mivacurium. Apart from perioperative complications, these patients are also more likely to suffer potentially life-threatening complications of cocaine toxicity [5]. Furthermore, recent work has posited low levels of serum pseudocholinesterase at birth as a vulnerability, and a potential biomarker, for sudden infant death syndrome [6]. The clinical significance of pseudocholinesterase deficiency therefore extends beyond the perioperative setting.

Suspected clinical diagnoses can be confirmed by laboratory tests. The dibucaine number indicates the percent of pseudocholinesterase activity that is inhibited by dibucaine [2]. It provides an indirect measure of pseudocholinesterase activity based on the differential patterns of inhibition exhibited by genetic variants compared with the wildtype [7]. Quantitative tests, such as serum cholinesterase activity, determine the amount of the enzyme in a blood sample. Finally, molecular testing using polymerase chain reaction (PCR) can identify specific variants in the *BChE* gene, providing evidence regarding the etiology of disease.

The patients’ parents have provided written consent for the publication of this case report.

## Case presentation

A healthy 18 kg 4-year-old girl of Northern European descent [American Society of Anesthesiologists (ASA) Physical Status Classification I] presented for laser tonsillotomy, adenoidectomy, and tympanocentesis. Premedication included oral midazolam (0.1 mg/kg) and ibuprofen (10 mg/kg). Following attachment of standard monitoring devices [electrocardiogram (ECG), non-invasive blood pressure, pulse oximetry], anesthesia was induced with intravenous fentanyl 20 µg, propofol 70 mg, and mivacurium 6 mg. Dexamethasone 2.5 mg and ondansetrone 2 mg were given for prophylaxis of post-operative nausea and vomiting. General anesthesia was maintained with remifentanil (0.5 µg/kg/minute) and propofol (10 mg/kg/hour) under continuous processed electroencephalogram (EEG) monitoring (Narcotrend, Narcotrend-Group, Hannover, Germany; target range 40–60).

**Fig. 1:**
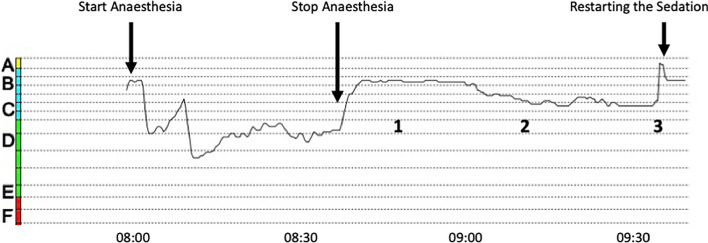
Narcotrend indices throughout the operation and perioperative period. After induction of anesthesia, the electroencephalogram index drops to D2 (40–60). Administration of anesthetics is stopped 5 minutes before the end of the operation. The index rises to B1–B2 (80–89) and remains at this level for about 20 minutes [1]. At this stage, spectral analysis shows a typical propofol pattern characterized predominantly by beta activity. Interestingly, the electroencephalogram index subsequently drops again (C1–2, 70–79), despite cessation of anesthetic administration [2]. Spectral analysis now reveals significantly reduced beta activity with an increase in theta waves. This pattern likely reflects the residual effect of propofol (beta waves) alongside the child’s sleep electroencephalogram (theta waves). A sudden increase in the electroencephalogram index to A (94) raises suspicion of a relaxant overhang, whereupon sedation is restarted [3]. When propofol is restarted, beta activity in the electroencephalogram increases again, and the index begins to fall

The immediate postoperative course was notable for a lack of spontaneous ventilation and delayed emergence, despite Narcotrend indices of B1–B2 (80–89)—indicating light residual sedation—approximately 25 minutes after discontinuation of anesthetics (Fig. 1). Interestingly, Narcotrend indices subsequently decreased again, providing readings of C1–C2 (70–79) over the following 40 minutes. A sudden spike to A (94), despite sustained apnea, prompted evaluation of her neuromuscular status and immediate re-initiation of sedation [remifentanil (0.2 µg/kg/minute) and propofol (4 mg/kg/hour)].

Recovery from neuromuscular blockade was evaluated using a peripheral nerve stimulator. Train-of-four testing (TOF, 2 Hz) did not elicit a response (TOF 0/4), indicating residual paralysis. A clinical diagnosis of suspected pseudocholinesterase deficiency was made after brief consideration of possible—but less likely—differential diagnoses (that is, narcotic overdose and central anticholinergic syndrome). Transfer to our pediatric intensive care unit was arranged for continued mechanical ventilation and monitoring.

The subsequent postoperative course was unremarkable. The patient could be safely extubated 8 hours after the administration of mivacurium (TOF 4/4), and was transferred back to the department of otorhinolaryngology. She was issued a medical alert card and discharged home after 2 days of observation.

The clinical diagnosis was confirmed using laboratory tests. Serum pseudocholinesterase activity was 940 kU/L (normal range 3200–6600 IU/L) [2]. Genetic testing identified compound heterozygosity in the *BChE* gene, and a diagnosis of autosomal recessive hereditary pseudocholinesterase deficiency was made. Compound heterozygosity is characterized by the presence of two different pathogenic alleles of a particular gene. Indeed, her non-consanguineous, phenotypically unremarkable parents were both found to be heterozygous carriers of different pathogenic variants of the *BChE* gene. Specifically, DNA sequencing identified a complex allele carrying the K and A variant of the *BChE* gene in the father (NM_000055.2: c. [293A > G; 1699G > A] p. (Asp98Gly; Ala567Thr); previously p. (Asp7OGIv:Ala539Thr)), while the mother was found to have the following variant: NM_000055.2:c. [428G > A; 1685-14 T > C] p. (Gly143Asp); previously p. (Gly115Asp) in).

## Discussion and conclusions

Mivacurium is a short-acting non-depolarizing muscle relaxant. It is usually rapidly metabolized by pseudocholinesterase, an hepatically synthesized plasma enzyme. The etiology of pseudocholinesterase deficiency can be genetic or acquired. Genetic mutations may affect the enzyme’s ability to efficiently metabolize mivacurium and succinylcholine [1]. Metabolization then relies on alternate pathways, such as nonspecific plasma esterases. Causes of an acquired deficiency include pregnancy [8], extensive liver disease [9], or malnutrition [10]. Interestingly, reductions in enzyme activity secondary to acquired causes or heterozygosity are not usually clinically significant, because enzyme activity rarely falls below 50%—the suggested threshold for symptomatic deficiency [8]. However, a combination of factors, such as pregnancy in a heterozygote, could potentially result in clinically significant reductions, causing postoperative apnea and paralysis—even in patients who may have previously undergone general anesthesia without anamnestic complications [2, 8]. It is therefore worth noting the different causes of pseudocholinesterase deficiency, and the possible interactions between them [2].

Genetic testing identified an autosomal recessive pattern of inheritance resulting in compound heterozygosity. Compound heterozygosity presents as functional homozygosity if both mutations cumulatively result in a clinically relevant decrease in enzyme activity. The patient’s parents, both of whom are phenotypically healthy and had previously undergone general anesthesia, were identified as heterozygous carriers of different mutant alleles of the *BChE* gene. The A and K variant detected in the father are relatively common mutations, affecting approximately 4% of the general population [2]. Given the autosomal recessive pattern of inheritance, the probability of the patient’s younger sister also being compound heterozygous for the *BChE* gene is 25%. However, at the time of submission, her genotype had not yet been determined.

We would like to emphasize three points that can be learned from this case. First, although rare, pseudocholinesterase deficiency should always be considered in the differential diagnosis of delayed emergence with sustained apnea. Moreover, one should be aware that a standard pre-operative workup will not flag pseudocholinesterase deficiency, and by definition, most cases will therefore present unexpectedly.

Second, evaluation of patients’ neuromuscular status using a peripheral nerve stimulator should routinely be performed following administration of mivacurium or succinylcholine. It is a simple, quick, reliable, and inexpensive way of ensuring that pseudocholinesterase deficiency will not be overlooked. It has also been shown to reduce the incidence of complications, such as awareness during emergence, thereby reducing the risk of distress and subsequent symptoms of post-traumatic stress disorder [11, 12].

Third, it may be helpful to monitor patients’ processed EEG until they have fully regained consciousness: a combination of increasingly light residual anesthesia, sustained apnea, and residual neuromuscular blockade will point to abnormal emergence, and is characteristic of pseudocholinesterase deficiency [13]. Thus, continuous “postoperative” processed EEG monitoring may help facilitate a timely diagnosis in cases of delayed emergence.

A timely working diagnosis of pseudocholinesterase deficiency is crucial, because it can help avoid unnecessary exposure to other medication (for example, naloxone, flumazenil, and physostigmine), and because the prognosis of pseudocholinesterase deficiency is excellent once supportive management has been initiated. Treatment of perioperative complications usually involves continued mechanical ventilation until the culprit agent has been metabolized and any residual paralysis has subsided. Interestingly, pseudocholinesterase activity is relatively stable in blood products and prolonged paralysis could be treated using transfusions of donor blood [14]. Furthermore, human serum cholinesterase could technically be used to accelerate recovery [15]. However, given the excellent prognosis under conservative treatment, the risks of blood transfusion need to be carefully balanced against the marginal benefit of reducing neuromuscular blockade by a few hours [1]. In contexts in which sufficient ventilators are available to provide ventilatory support until extubation is safe, the conservative treatment therefore remains the treatment of choice [1, 2].

In summary, pseudocholinesterase deficiency is a rare disorder that commonly presents as prolonged apnea and paralysis following general anesthesia after induction with succinylcholine or mivacurium. A causal treatment does not currently exist. However, the disorder typically has a benign course, and an excellent prognosis, if supportive management, including prolonged post-operative ventilatory support, can be provided. To ensure a timely diagnosis, pseudocholinesterase deficiency must be included in the differential diagnosis of delayed emergence, and the combination of sustained paralysis despite indicators of increasingly light residual sedation or distress (that is, increasing EEG indices, tachycardia, and hypertension) should immediately raise suspicions of an abnormal response to mivacurium or succinylcholine. Once suspected, a clinical diagnosis can be supported using a peripheral nerve stimulator, and confirmed using laboratory tests, while genetic testing can help determine the etiology of disease.

## Data Availability

All data generated or analyzed during this study are included in this published article (and its supplementary information files).

## Abbreviations

ASA: American Society of Anesthesiologists
BChE: Butyrylcholinesterase
ECG: Electrocardiogram
EEG: Electroencephalogram
PCR: Polymerase chain reaction
TOF: Train-of-four
